# Structural Differences Observed in Arboviruses of the Alphavirus and Flavivirus Genera

**DOI:** 10.1155/2014/259382

**Published:** 2014-09-16

**Authors:** Raquel Hernandez, Dennis T. Brown, Angel Paredes

**Affiliations:** ^1^Department of Molecular and Structural Biochemistry, North Carolina State University, Raleigh, NC 27695, USA; ^2^U.S. FDA/National Center for Toxicological Research, Department of Health and Human Services, Jefferson, AR 72079, USA

## Abstract

Arthropod borne viruses have developed a complex life cycle adapted to alternate between insect and vertebrate hosts. These arthropod-borne viruses belong mainly to the families Togaviridae, Flaviviridae, and Bunyaviridae. This group of viruses contains many pathogens that cause febrile, hemorrhagic, and encephalitic disease or arthritic symptoms which can be persistent. It has been appreciated for many years that these viruses were evolutionarily adapted to function in the highly divergent cellular environments of both insect and mammalian phyla. These viruses are hybrid in nature, containing viral-encoded RNA and proteins which are glycosylated by the host and encapsulate viral nucleocapsids in the context of a host-derived membrane. From a structural perspective, these virus particles are macromolecular machines adapted in design to assemble into a packaging and delivery system for the virus genome and, only when associated with the conditions appropriate for a productive infection, to disassemble and deliver the RNA cargo. It was initially assumed that the structures of the virus from both hosts were equivalent. New evidence that alphaviruses and flaviviruses can exist in more than one conformation postenvelopment will be discussed in this review. The data are limited but should refocus the field of structural biology on the metastable nature of these viruses.

## 1. Background

### 1.1. Arbovirus Evolution

The arboviruses are not a taxonomic classification, but rather a grouping based on viral transmission through an insect vector to infection of a vertebrate host. The arboviruses contain members of the Togaviridae, Flaviviridae, Bunyaviridae, Rhabdoviridae, Reoviridae, and Orthomyxoviridae and are also represented by a single DNA virus, African swine fever virus family Asfarviridae of genus Asfivirus http://ictvonline.org/virusTaxonomy.asp. Evidence exists that arboviruses from the alphavirus lineage evolved from plant viruses [1, 2] which adapted to growth in insects [3]. Hematophagous insect viruses then acquired the ability to infect vertebrates, thus adapting from separate kingdoms (plant to insect) as well as phyla (insect to vertebrate) [4]. Members of the Bunyaviridae still maintain the plant to insect cycle [5–7] as well as the insect only cycle [8–10]. Arbovirus members of the flaviviruses are believed to have emerged about 1000 years ago in a nonhuman primate to mosquito cycle [11, 12] from predecessors that date at least 85,000 years [8]. It has been suggested that each of the 4 dengue serotypes (DEN1-4) adapted to humans independently only a few hundred years ago [13]. It is believed that this capability to diversify so broadly must have arisen from the inherent error-prone nature of the RNA-dependent RNA polymerases [14] while also limiting the evolution of arboviruses to certain families within the RNA virus class that are highly error-prone [14–16]. It is thought that the ability of viruses from each of these families to use or infect vertebrate hosts arose independently [17]. For these viruses to be able to cycle between insect and vertebrate hosts, their genomes must be compatible to hosts of two divergent phyla. This has been achieved by the evolutionary selection of virus that represents a consensus sequence able to function in both hosts. Thus, the arboviruses represent genomes selected by multiple mechanisms of adaptation and are exposed to repeated selection.

For the families Togaviridae, Flaviviridae, and Bunyaviridae, which comprise the bulk of arboviruses, the structure of the glycoprotein E1, E, and possibly Gc, respectively, appear to have arisen from an ancient predecessor [3]. While the sequences of the E1 cognate glycoproteins have diverged in the Togaviridae, Flaviviridae, and Bunyaviridae, the function and structures of these viruses have been retained [18]. Evolution of the protein structure has been constrained by adapting to both arthropod and vertebrate hosts. This difference in the rates of genomic divergence has been seen in a nonarbovirus member of the Togavirus family, Rubivirus [19] in which the known structure of E1 appears to have diverged relative to the arbovirus members of this family [20]. This observation suggests that the consensus sequence of the arboviral genome is maintained by eliminating genetic drift, which impacts fitness in each host. In other words, the virus sequence evolves more slowly when divergent hosts are continuously selecting for virus fitness. Collectively, the available information suggests that the mosquito-borne viruses acquired the form we now see from the arthropod vectors and did so concurrent with becoming hematophagous, presumably to optimize egg maturation [21].

### 1.2. Arbovirus Structure

**Figure 1:**
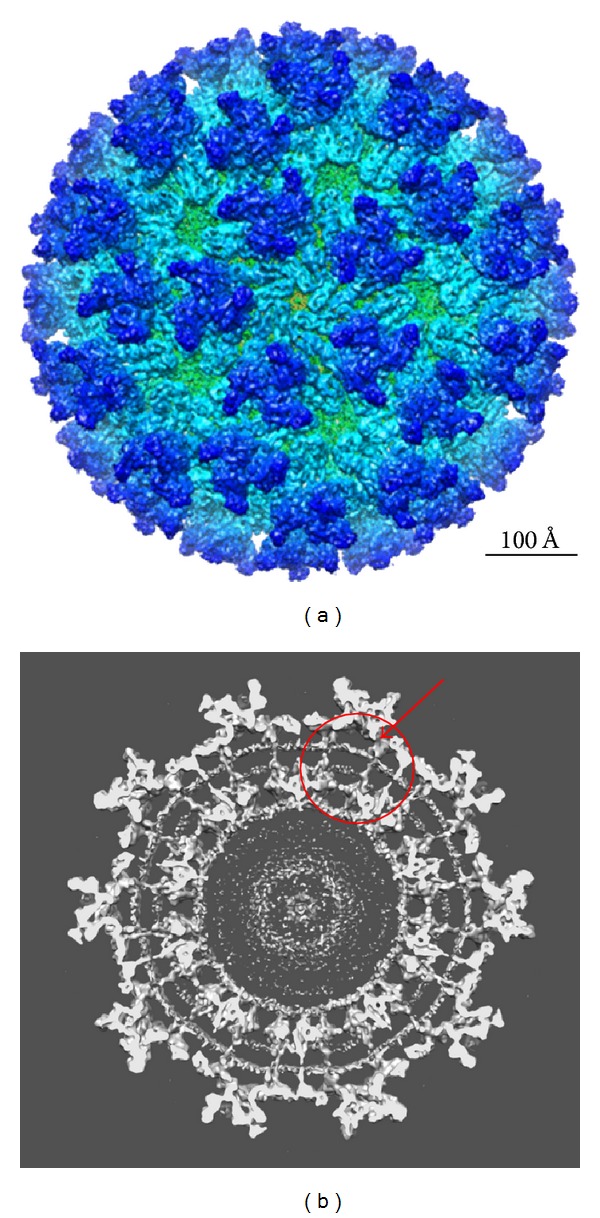
Shown in (a) is the cryo-EM reconstruction of the TC-83 strain of Venezuelan equine encephalitis virus (VEE) viewed down the strict 5-fold axis. Resolution is 4.4 Å. The trimers consist primarily of E2 with the smoother “skirt” comprised of E1. In (b) is shown a slice down the 2-fold axis. Note the transmembrane domains (arrow) and the extensive organization of the nucleocapsid (circle) compared to that seen in the flaviviruses (Figure 3(b)). With permission from Zhang et al. [22]. For full resolution images see EM DATA BANK (EMDB)/5275.

Of the seven families of arboviruses, three (Togaviridae, Flaviviridae, and Bunyaviridae) are icosahedral, membrane-containing plus-stranded RNA viruses. It is interesting that though the respective glycoproteins E1, E, and Gc of the alpha, flavi, and bunyaviruses encode the same basic protein fold, these proteins assemble into different icosahedral structures. Alphaviruses are *T* = 4, (Figure 1) [22], flaviviruses are *T* = 3 (Figure 3) [23], and bunyaviruses (*Phlebovirus*) are *T* = 12 [24, 25]. This capability of viruses to assemble an inherently similar global fold into their different structures is dependent on the specific functions of their structural proteins. Rhabdoviruses are enveloped but assume a rod or bullet shaped structure that demonstrates some helical symmetry [26]. Reoviruses (*T* = 13 l, laevorotatory, turning toward the left) are icosahedral but do not contain a membrane [27]. Orthomyxoviruses are enveloped viruses that display pleomorphic structures ranging from spherical such as influenza A to filamentous as in influenza C [28]. In those influenza structures assuming a spherical shape, that shape is not due to icosahedral symmetry. In addition, orthomyxoviruses have more lipid-membrane relative to their envelopes than do enveloped icosahedral viruses resulting in the pleomorphism displayed [29]. The Asfarviridae viruses are large double-stranded DNA viruses, spherical to pleomorphic, and 175–215 nm in diameter and exhibit icosahedral symmetry (*T* = 189–217). These viruses are composed of two icosahedral protein shells that contain an intervening lipid structure [30]. These structural differences illustrate that there is no common virus structure adopted by the arboviruses which by itself would explain how these viruses infect both arthropods and vertebrates. There are two systems of virus classification currently in use. While morphology is a useful basis for virus identification and classification, at present, two classification systems exist. The hierarchical virus classification system, the International committee on taxonomy of viruses (ICTV) [31], and the Baltimore classification system. The Baltimore classification system is based on nucleic acid type which places viruses into seven groups in hierarchical classification [32]. The ICTV uses evolutionary relationships as the classification scheme [33]. ICTV classification places viruses by phenotype; morphology, protein composition, and genotype; nucleic acid type, sequence, mode of replication, host organisms infected, and the type of disease they cause. Capsid structure has also been utilized to organize virus groups based on the hypothesis that only a limited number of protein folds are available to self-assemble a nucleocapsid [24, 34]. This review will be limited to the group arboviral members of the alphaviruses and flaviviruses for which considerable structural information exists. Bunyaviruses are less well studied and are proposed to be functionally analogous to flaviviruses by computational, proteomic, and indirect biochemical analysis [35]. Studies on whole virus particles will be the focus of this discussion.

### 1.3. Alphaviruses and Flavivirus Virion and Genome Structure

**Figure 2:**
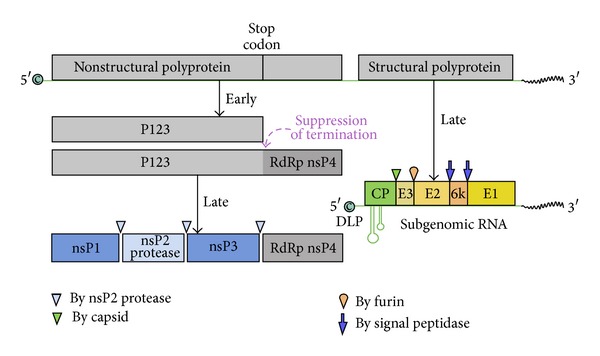
Alphavirus genome organization. Alphaviruses are organized with the (nonstructural) ns proteins at the 5′ end of the genome The ns proteins are translated from the genomic RNA while the structural proteins are translated from a subgenomic RNA that includes the 3′ end of the genome. The RNA polymerase nsP4 is a read-through protein present early during infection. After replication is established, protein production switches to the structural proteins C, E3, E2, 6K, and E1. The C protein autoproteolytically cleaves itself from the remaining polyprotein. Only E1, E2, and C are found in the mature virion. In some cases, E3 may also be associated with the virus particles [Virus DB: VBRC genome browser accession VG0000908].

The alphaviruses are a genus in the family Togaviridae. They are icosahedral viruses of *T* = 4 geometry and contain a +polarity single-stranded RNA genome. The genome is organized with the 4 nonstructural genes found at the 5′ end of the capped RNA genome followed by the structural proteins capsid (C), PE2, 6K, and E1 (Figure 2  [Virus DB: VBRC genome browser accession VG0000908]). The virus is composed of the structural proteins C, E2, and E1 that are synthesized from subgenomic RNA made from an internal promoter [36]. This RNA is then translated as a polyprotein and processed by viral and host enzymes during maturation and comprises the virion. The glycoproteins are assembled within the ER. The 5′ capsid protein is autoproteolytically processed from the proprotein and organizes the genomic RNA into a nucleocapsid. During virus maturation, PE2 is converted to E3 and E2 by furin [37]. In most cases E3 does not remain associated with the virus [38]. E1 and E2 form trimers of heterodimers that envelope the nucleocapsid which assembles independently [39]. By regulating RNA and protein synthesis in a temporal manner, the Sindbis virus is able to quickly replicate to as many as 10^6^ particles/cell [40]. Alphaviruses bud from the cell surface in mammalian cells but are assembled in vacuoles in mosquito cells and mature via the exocytic pathway [41]. These viruses are hybrid in nature acquiring lipids, carbohydrates, and other modifications from the host cell while their proteins are virus encoded. The final assembled structure is that of two nested spheres separated by an intervening membrane bilayer held together by protein associations between the E2 protein endodomain and the capsid protein.

Flaviviruses are in the family Flaviviridae and are also membrane-containing viruses, but they assemble into pseudo *T* = 3 icosahedra. The genome is organized with the structural proteins at the 5′ end of the +strand RNA molecule and is translated into a single polypeptide which is processed during maturation by viral and host-encoded enzymes into multifunctional proteins. The three structural and seven nonstructural proteins are cleaved by a series of virus and host-encoded proteases. The structural proteins starting at the 5′ end of the monocistronic genome include C, preM, and E (Figure 4 [Virus DB: VBRC genome browser]). As with the alphaviruses, the flavivirus polyproteins are inserted into the ER of the host cell. However, the flaviviruses are also assembled in the (endoplasmic reticulum) ER with concurrent assembly of the nucleocapsid RNA/C structure [42]. preM and E form dimers during protein maturation resulting in further processing of preM to M as the glycoproteins mature [43]. Little is known about the process of encapsidation into the mature virion, but incorporation of the nucleocapsid of these viruses is thought to involve nonstructural proteins [42]. Flavivirus particles differ from alphavirus particles in that the nucleocapsid association is more peripheral to the intervening membrane, and no organized structure is observed [42, 44]. The particle structure is also nested with an intervening membrane, but no strong contacts with the M and E membrane domains are formed [45, 46].

## 2. Review

### 2.1. The Plasticity of Alphavirus Variants as Seen by Ultrastructure

The alphavirus virion assembles into a stable structure that shields the genome from the adverse effects of the surrounding environment. Virus particles are assembled into a high energy state in which infectious particles are poised to deliver their genomic cargo after the appropriate stimuli are encountered through specific interaction(s) with the host cell. Thus, the infectious virion is a metastable intermediate that assumes sequentially different conformations depending on the pH, temperature, and host environments it encounters (discussed below). The plasticity of the virion structure underscores the flexibility of the viral proteins. This flexibility is most likely not due to global changes in the structural proteins but rather local changes in the metastable domains of the proteins [48]. For structural stability, the virion as a whole must contain protein domains with structural stability imparted by the protein sequence itself or via stabilizing protein-protein interactions. We describe two examples of the alphavirus Sindbis laboratory constructed mutants producing structural variants that illustrate the ability of point mutations in the structural proteins to acquire novel architecture.

The first example is that of the Sindbis virus capsid protein mutant Y180S/E183G. Sindbis virus is a macromolecular structure composed of RNA, protein, and lipid. The inner protein shell, the nucleocapsid, is bound to the outer protein shell via interactions to the E2 endodomain. Genetic and structural evidence suggest that the nucleocapsid interacts with the E2 endodomain by aromatic amino acid interactions between Y420 in the E2 protein and Y180 and W 247 in the capsid protein [49, 50]. Mutations in the capsid protein, Y180S/E183G, result in the assembly of the virus structural proteins into icosahedra of increasing triangulation numbers. The triangulation numbers calculated for these morphological variants, follow the sequence *T* = 4,9, 16,25, and 36 [51–53]. All fall into the class *P* = 1 of icosadeltahedra. It has been suggested that the* T* = 4 structure of the nucleocapsid organizes the outer glycoprotein layer [47, 54]. These observations support models suggesting that the geometry of the preformed Sindbis nucleocapsid organizes the assembly of the virus membrane proteins into a structure of identical conformation. It has been proposed that these two mutations in the capsid protein endow the protein with the flexibility to increase the number of capsid proteins incorporated into the nucleocapsid before formation of the next five-fold vertex. This is seen more readily in the smaller icosahedra, examples of which are shown in Figure 5. The structures of increasing size and triangulation number also form in mosquito C7-10 cells suggesting that the capsid protein mutation and not the host cell or the temperature of assembly is involved in the expression of this phenotype (unpublished data). This mutant demonstrates the capability of a capsid protein fold to “evolve” a new triangulation number and structure. These larger capsid structures can package large RNA molecules [55]. While the structures formed by this mutant are not stable, they are infectious and it is possible that subsequent mutations could stabilize any of the resulting new icosahedra (Figure 6).

A second example of a Sindbis virus structural protein mutant able to organize into nonnative structures is that of the furin mutants in the glycoproteins E1 and E2 shown in Figure 7 [53]. Furin protease recognition sites, Arg-X-Arg/Lys-Arg, were installed into the E1 sequence at amino acid positions 392 and E2-341. The numbering scheme refers to the position of the first mutated Arg to create the furin site. The furin double mutant was found to produce virus particles of normal infectivity and structure when grown in mammalian cells (particles composed of the requisite number of wild type proteins, see [37]) (Figure 7(A)) as well as virions that developed long tubular appendages of varying lengths (virions incorporating aberrant proteins) [53]. Virus production from insect cells was of insufficient titer to allow microscopic examination. The tubular structures are 73 nm in diameter, consistent with the size of the wild type virion. Scanning electron microscopy of double mutant infected cells revealed that maturing particles were initiated with spherical structures that probably contained a capsid (Figure 7(B) arrow). The large amorphous structures as seen in Figure 7(K) may contain multiple capsids with associated membrane. It was concluded that the tubular structures initiated by organizing around the icosahedral nucleocapsid and depending on the number of furin processed proteins incorporated, terminated the envelopment process by normal membrane fission (budding) or by repeated incorporation of hexagonal protein arrays. In the absence of the quasi-equivalent protein interactions of the icosahedral five-fold vertex, the only lattice that can form is a hexagonal sheet in one dimension or a hexagonal tube in 3 dimensions. The geometry of the tubular helices was determined by calculating the optical contrast reinforcement of the EM images of particles such as those seen in Figure 8. Thus, when densities are in phase, they will reinforce one another and a repeating distance can be calculated. This was seen at 16 and 20 nm, distances consistent with the spacing of the wild type virus 6-fold symmetry, shown in Figure 9, indicating that the geometry of the mutant particles is relevant biologically. For the furin mutant E1-392/E2-341, we see that a loss of function mutation in the structural glycoproteins E1 and E2 can result in the ability of the virion to maintain the protein-protein interactions that enable the formation of a helical array. Again, the flexibility of these viral proteins to produce variant structures underscores the plasticity of these proteins and illustrates the ability of viruses to explore possible new structures to enable efficient survival and facilitate evolutionary divergence.

These mutant virus structures help us to understand the metastable structure of alphaviruses by illustrating that assembly of the virion proceeds through structural intermediates. In the case of the C mutant, this protein can fold into native or the mutant conformations and assemble multiple forms. For this change to occur, the mutant protein must be able to assume an energetically stable intermediate. This is thought to happen during the folding of the protein and assembly, in this case, of the nucleocapsid. The E1-392/E2-341 mutant demonstrates that the metastability of the virus extends to both shells and can trap the glycoproteins into a conformation that only allows the formation of the hexagonal lattice.

### 2.2. Protein Structure

Protein conformations have been reported for many of these virus structural proteins as single proteins and in protein aggregates (http://www.ncbi.nlm.nih.gov/). The metastable state of the virion, however, suggests caution in interpreting structures of the isolated structural proteins. Virus particles are macromolecular machines poised to deliver their genomic cargo once the appropriate stimuli are encountered. These particles must also shield the genome from the adverse effects of the surrounding environment. These requirements are accomplished by the virion assembling into intermediates which refold into a highly energy rich infectious conformation, which is a sufficiently stable structure [56]. Thus, the infectious virion is a metastable intermediate that can exist in more than one conformational state depending on pH, temperature, and the complex host biochemical environments (discussed below). With the correct stimulus, such as interaction with a receptor, the virus undergoes a conformational change that is propagated through the virion to initiate infection. Not all virions are infectious because the structural integrity of the particle is compromised during assembly or virus preparation. Thus, in a field of virus particles the number of noninfectious particles can outnumber infectious particles by as many as 1000 to 1. This large particle/pfu ratio can only complicate study of the native, infectious structure. Only stable or transiently stable conformations of these “metastable” proteins can be crystalized or imaged by cryoEM as was done with flu virus [57–59]. The isolated flu structural proteins comprise limited conformations of very flexible proteins with multiple functions. While crystal structures for Sindbis and Chikungunya virus structural proteins have been reported, these may not be native because of the lack of the transmembrane domain and many more conformations of infectious intermediates may exist for these viruses. This is evident from both quasiequivalence and from the different conformations of E1 that have been identified in Sindbis virus [56, 60–66]. It has been reported that, during Sindbis virus infection, disulfide exchange may occur to enable the formation of the fusogenic conformation [67] and is important for the proper assembly of virions [56, 68]. The conformational changes induced by disulfide exchange during infection are not understood but are known to be required for infectivity of the virus [66]. Although there is no independent evidence to verify that the crystal structure of E1 is a native structure, there is independent evidence that different forms of E1 exist in the alphavirus structure when whole infectious virus is analyzed [63]. The argument that the crystal structures “fit well” into the cryoEM electron density maps gives much leeway to the interpretation of the structure. Because of quasiequivalence, a single high resolution X-ray crystal structure frequently cannot be fitted into a cryoEM electron density map of a high *T*-number virus without distorting the X-ray structure in order to make the fit. For Sindbis virus, a single alphavirus E1 structure is commonly used in virion structural reconstruction analysis, at least four other conformations of the E1 protein are known to exist as the protein is folding and unfolding [56, 60, 69]. Structural intermediates of disulfide bridged proteins that are detected on nonreducing PAGE after extraction of the E1 protein from infectious virus also form multiple intermediates [56]. During alphavirus glycoprotein maturation, E1 and PE2 form integrated trimers of heterodimers. E2 is a molecular scaffold as well as the escort protein that delivers multimers to the cell surface [69]. Mass spectroscopy analysis of cysteines found in infectious virus was not the same as those reported for the alphavirus E1 crystal [66]. For these reasons it is critical to use infectious virus to assess structure because alphavirus E1 infectious conformations only exist in the intact, metastable virus [66]. Stated succinctly, native intermediates of E1 cannot exist in solution because cysteines will reassort as the high energy form becomes stable. While whole virus analysis has proven difficult because the resolution of most whole virus reconstructions is not at atomic levels even when the resolution is increased by “fitting” a crystal new technology can address these problems.

The cryomicroscopes of today include new advancements such as a phase plate used to negate the contrast transfer function (CTF) of the microscope so that images do not require CTF correction. The phase plate technology is still under development because those that work well contaminate quickly when used [70–73]. Additionally, Charged-couple device (CCD) cameras are being replaced with direct (electron) detectors. These detectors capture hundreds of images per second and once captured, the images are averaged together to reduce noise and to correct for specimen drift to boost both resolution in the image and signal. As a consequence, data are now being collected that are used to determine structures of biological samples below 4 angstroms resolution. This is close enough to atomic resolution that the carbon backbone of amino acids, including the R-side chains, can be followed in the structure. Depending on size, this technology will eliminate the need for pseudocrystal structures for biological samples [74, 75].

**Figure 3:**
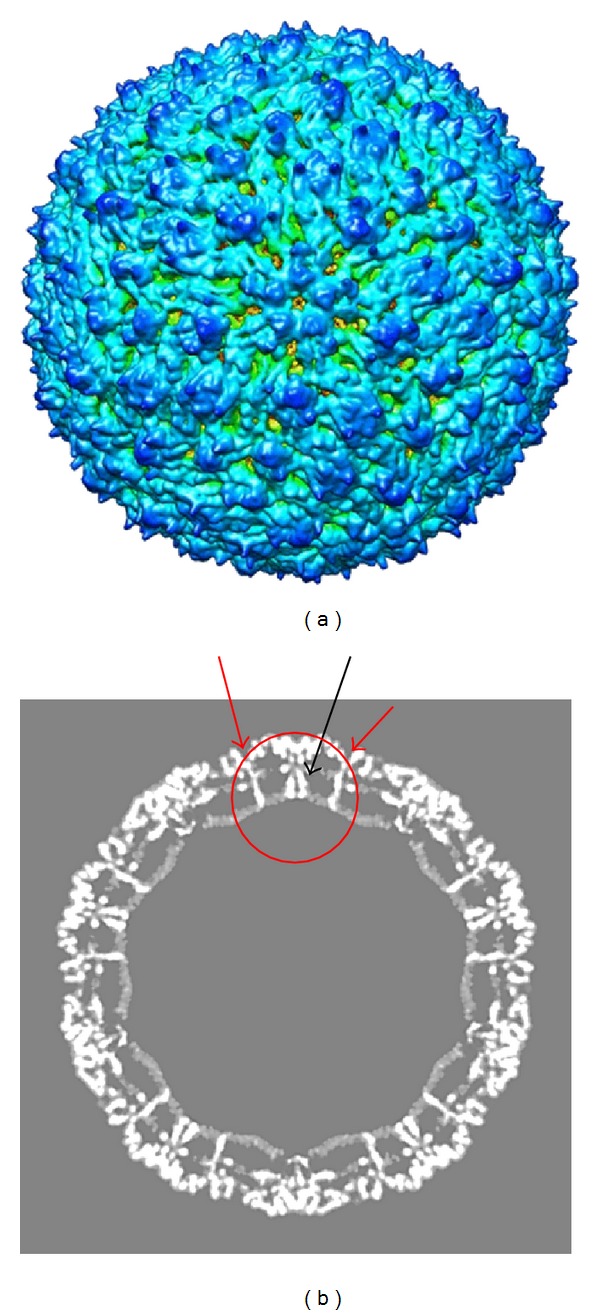
(a) Dengue 2 New Guinea C cryoEM reconstruction rendered at 3.5 Å, EMD 5520. The view is down the icosahedral 5-fold axis. Although this is termed a mature virus because of the smooth appearance, preM may still be associated with the virus. The surface of the structure has few distinctive features; however, it has been proposed that, upon maturation, the virus E protein undergoes major conformational changes as infection is initiated. (b) This image is a slice of the virus down the 2-fold axis. M (black arrow) and E (red arrow), transmembrane domains are seen below the outer surface. Distortion of the lipid membrane is seen where the transmembrane domains penetrate and no organized capsid structure is detected (circled). Compare to the alphavirus slice in Figure 1 where the nucleocapsid structure is clearly delineated. Reprinted with permission from Zhang et al. [23]. See EM DATA BANK (EMDB)/5520 for full resolution images.

**Figure 4:**
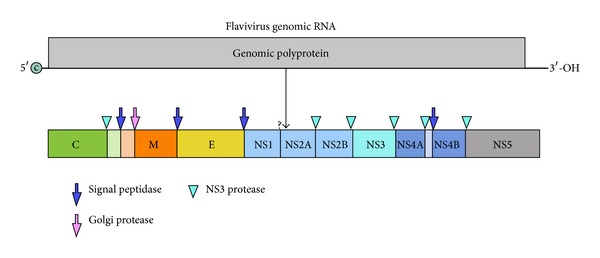
The 5′ terminus of the genome is capped and the polyprotein is expressed as a single polypeptide. The 3′ terminus is not polyadenylated but rather forms a loop structure. The locations of the nsP and structural proteins are switched with respect to the 5′ end of the RNA compared with the alphaviruses. It has been proposed that this switch occurred via recombination in an ancient precursor, thus retaining the structural glycoprotein folds. Capsid protein, preM, and E proteins are proteolytically processed as indicated by the enzymes shown by the arrows and arrowheads [Virus DB: VBRC genome browser].

**Figure 5:**
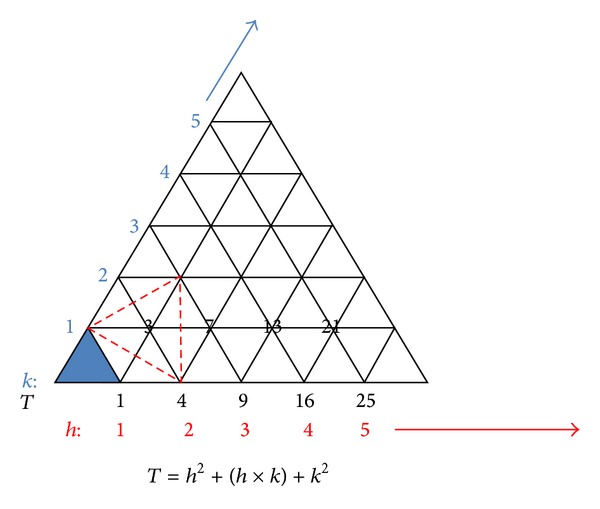
A family of icosahedra. Shown at the bottom of the large triangle, in blue, are the members of the *P* = 1 icosahedra. The triangulation number represents the number of non- or quasiequivalent structural morphological units per asymmetric unit of the icosahedral face. *T* = 1 is the only structure with a single equivalent morphological unit per icosahedral face, shown in blue. Neighboring 5-fold vertices of the 3-fold triangular face are connected to one another by three 2-fold axes giving the structure an overall 5-fold, 3-fold, and 2-fold symmetry. The *T* number, also determined by the formula *T* = *h* ^2^ + (*h* × *k*) + *k* ^2^, indicates the relative position of each 5 fold vertex as a function of the number of quasiequivalent morphological units per icosahedral face. Sindbis, with four quasiequivalent conformations of the E1E2 heterodimer, is a *T* = 4 structure. In the capsid mutant Y180S/E183G, the flexibility of the capsid protein is altered allowing the mutant protein to adopt a wider range of quasiequivalent conformations that increases the triangular number of the structure. Superimposed on the *P* = 1 lattice is a class 3 triangular face outlined in red. Flaviviruses are arranged in this *T* = 3 symmetry and belong to the class *P* = 3 icosahedra shown in black on the *k* = 1 axis.

**Figure 6:**
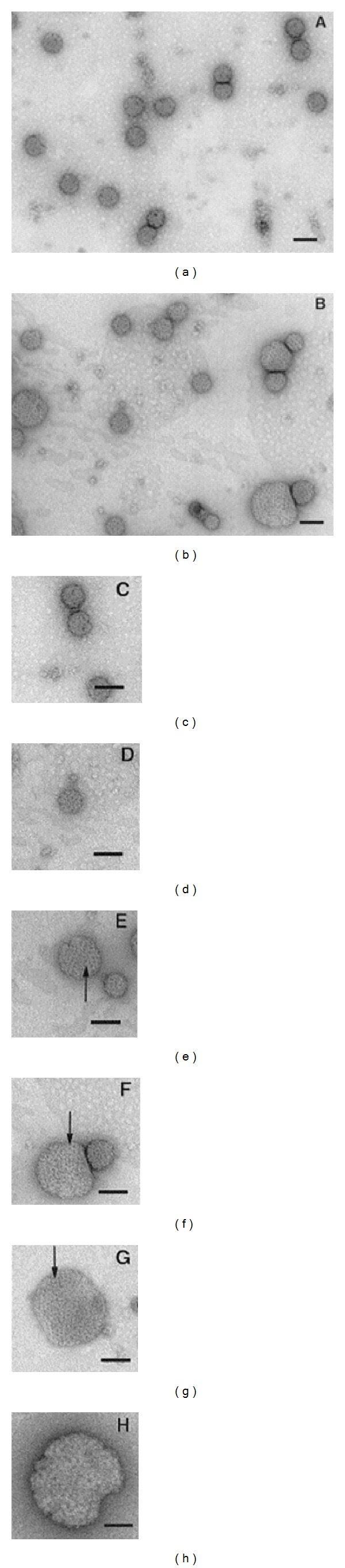
Electron micrographs of negatively stained virus particles recovered from the supernatant of BHK cells transfected with either Sindbis virus S420Y ((a) and (c)) or Y180S/E183G ((b), (d), (e), (f), (g), and (h)). (a) and (b): a wider view of the fields. (c)–(h): individual particles arranged to show examples of the various sizes found for the Y180S/E183G mutant ((d)–(h)) as compared to the control S420Y particle (c). Arrows point to morphological units. Bars, 100 nm. Ferreira et al. 2003 [47].

**Figure 7:**
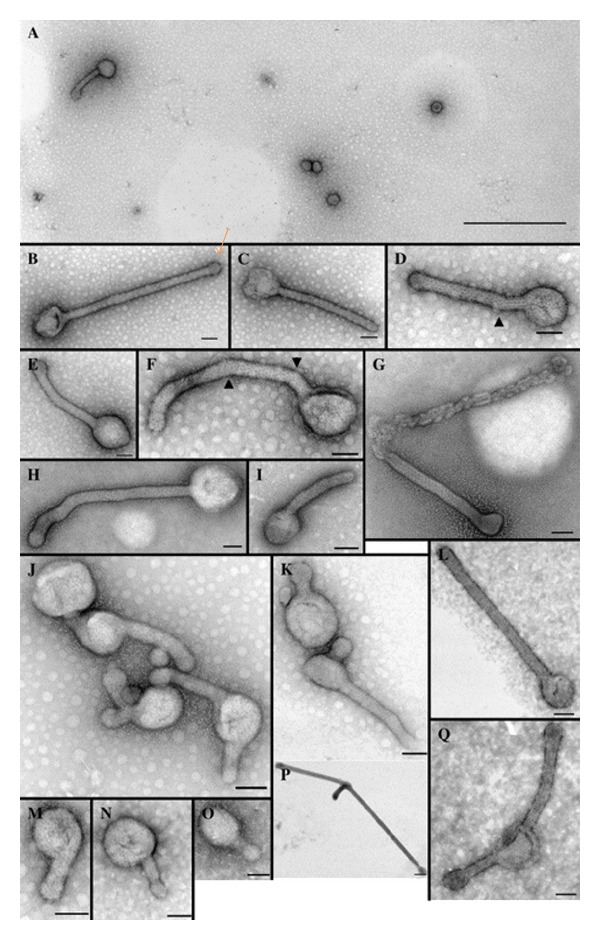
Electron micrographs of negatively stained furin sensitive double mutant E1-392/E2-341 containing virus recovered from the supernatant of transfected BHK-21 cells. (A) Low magnification of a typical sample of double mutant E1-392/E2-341. Bar, 1 *μ*m. ((B)–(Q)). Selected particles demonstrating the variations seen in one preparation of E1-392/E2-341. Arrowheads show points of helical disruption and reinitiation. Bars, 100 nm. Kononchik et al. 2009 [53].

**Figure 8:**
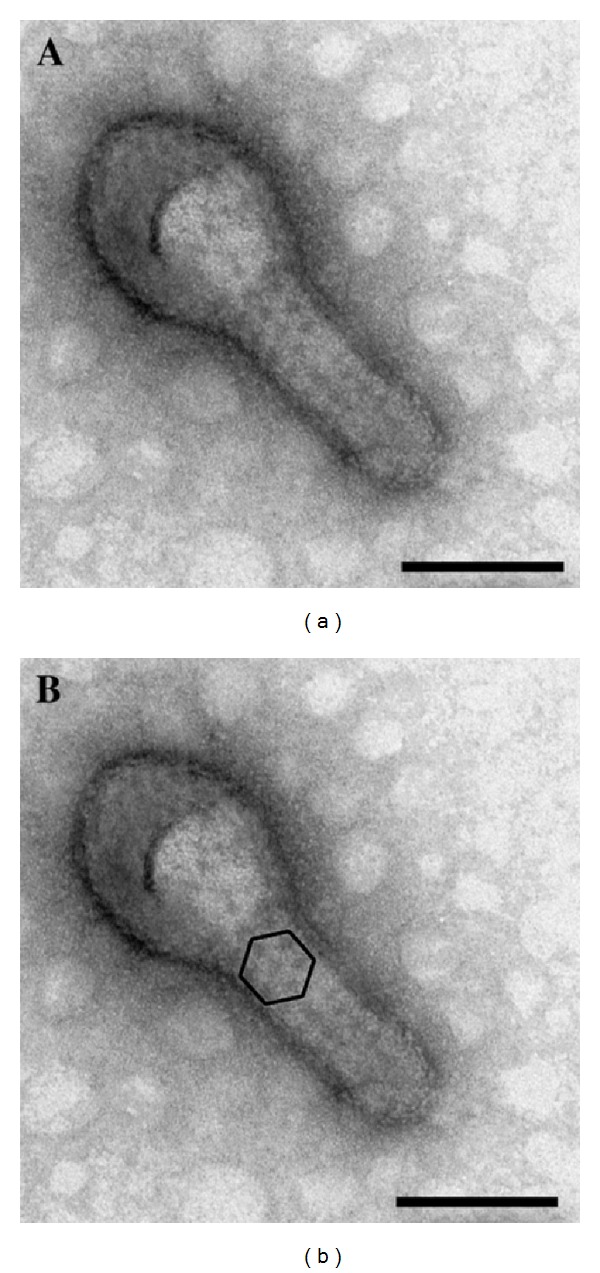
Electron micrograph of a negatively stained furin double mutant E1-392/E2-341. (a) A normal E1-392/E2-341 particle that shows surface detail. (b) The same particle (a) highlighting a hexagonal array clearly visible on the tubular structure. Bars, 100 nm. Kononchik et al. 2009 [53].

**Figure 9:**
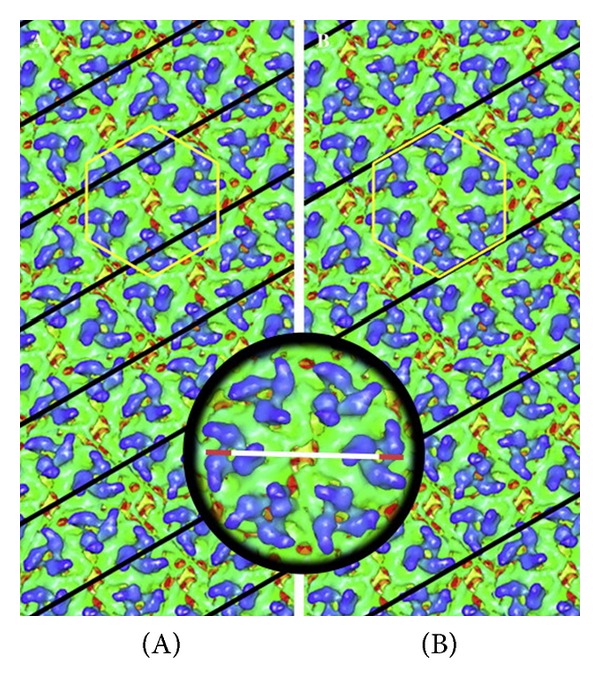
Helical reconstruction of the tubular section of the furin sensitive E1-392/E2-341 created by 6-fold rotational arrays (highlighted). Black lines illustrate the 16 nm repeat (A) and the 20 nm repeat (B) registers seen on the tubular section of the mutants. Illustrations are drawn to scale with the average width of the tubular structures. The insert illustrates the distances taken from a cryoelectron microscopy reconstruction of wild-type Sindbis virus measuring across a strict 2-fold axis of a hexagonal array: white is 16 nm and white with red is 20 nm. Kononchik et al. 2009 [53].

As seen in Figure 3(b) [23] flaviviruses are more fragile and therefore display a larger particle to pfu ratio than alphaviruses [76, 77]. This is the result, in part, from the weaker association of the nucleocapsid with the structural proteins. Organization of the RNA within the C protein is unclear. How the nucleocapsid interacts with the glycoproteins preM and E during assembly is also vague [78, 79]. No nonstructural proteins are incorporated into the virion although nonstructural proteins have been implicated in encapsidation and budding of the virus from the ER [80, 81]. The preM protein functions as a chaperone during E folding [42, 82, 83]. During transport of the virion to the cell exterior, preM is cleaved by a furin-like protein resulting in virus particles with M protein in its mature form [43, 84]. The processing of preM to M by furin is not as efficient as that seen for the alphavirus PE2 protein processed to E2 and E3; thus, particles with preM are detected [85, 86]. It has been shown that the lack of preM results in poor protein folding and poor immunogenicity [87]. Because E is the primary a immunogen, this implies that the native conformation of the E protein can be compromised when expressed* in vitro* or outside the context of the virus. Soluble E protein X-ray structures have been solved for many of the flaviviruses [88–91]. The structure is divided into 3 domains, I, II, and III [92]. Domain I is the N terminal portion of the protein and is centrally located within the crystal structure. Domain II contains the fusion peptide and the dimerization domain, while domain III is an immunoglobulin like domain and is thought to contain the receptor binding site [93]. This 3D structure is similar to that of the alphaviruses with analogous functional domains [94]. Unlike the alphavirus E1 protein, the flavivirus TBE and dengue E protein crystalized into protein dimers [95]. E protein from WNV is crystalized as a monomer but is fit into cryoEM as a dimer [96]. It is widely held that flavivirus E protein is induced by low pH to reorganize into a fusogenic trimer which initiates infection from an acidified endosome. While much indirect evidence has been reported to support this model of infection for alpha and flaviviruses, by comparison to flu, [92, 97–99], a second model of direct penetration by the virus at the host cell surface, determined by direct observation, is largely dismissed in favor of the fusion model. Ideally, a working model of Togavirus penetration should address all experimental evidence. Two issues will be discussed, first the use of indirect evidence from structural models which describe a fusion pathway and second, direct observation by ultrastructural and biochemical analysis that provide empiric evidence that infection by these agents is direct penetration at the plasma membrane.

### 2.3. Electron Cryomicroscopy

As electron optics and electron cryomicroscopy in general have improved, it has become possible to take images of frozen hydrated viruses from electron microscopes and use them to reconstruct the three-dimensional structures of infectious virus to ever higher resolutions by cryoEM (reviewed in [100]). Electron cryomicroscopy or cryoEM has allowed the placement of the different structural components of the glycoprotein shells of arboviruses for which structural data is available. The alphavirus Semliki Forest virus was first imaged by Vogel et al. [101, 102]. In 1987, Fuller reported the first cryoEM structure of Sindbis virus to be a *T* = 4 icosahedron surrounding a *T* = 3 core [103]; however, the core was later shown to be *T* = 4 [104]. The first cryoEM image of Sindbis virus with sufficient resolution to image the trimeric spikes was produced in 1993 by Paredes et al. [104]. This reconstruction confirmed a structure that had previously been postulated genetically and biochemically, [105–107]. The most outwardly protruding structure in the cryoEM image was a 3-fold trimer with laterally associated proteins. It was not possible at that time to identify which proteins corresponded to what part of the structure, although it was known that the virus particles were held together by an E1-E1 protein lattice [106, 108]. Several studies have since used cryoEM to study the structure of E2 in alphaviruses. In 1995, Cheng et al. reported their cryoEM reconstruction of Ross River virus, in which the authors concluded that the capsid protein bound as a monomer to the E1-E2 dimer in a 1 : 1 stoichiometry [109]. The alphavirus E2 density was also probed using Ross River virus and anti-Ross River E2 neutralizing FAbs that blocked attachment. In these cryoEM structures, the E2 FAb labeled E2 on the outermost density of the tip of the bilobed spike protein [110]. The position of E2 was later confirmed in a 1998 study by Paredes et al., when a mutant of Sindbis virus defective in processing the N-terminal E3 protein from the PE2 precursor was reconstructed using cryoEM. The reconstruction identified the additional E3 density at the tips of the trimeric spikes and identified the general locations of E2 in the spike region with E1 involved in the protein lattice at the base of the spike [111]. The general location of both proteins was reported in 2001 by deleting the carbohydrate modification sites from E1 and E2 singly and together in several nonglycosylated mutants. By comparing the cryoEM densities of the nonglycosylated mutants to wild type virus, it was possible to determine the relative positions of E1 and E2 on the virus surface [112].

In a 1990 study by Flynn et al., conformational changes in Sindbis virus E1 and E2 were observed as virus engaged the plasma membrane at neutral pH in cells that were not acidified [113]. These rearrangements were detected by (monoclonal antibody) MAb and corresponded to transitional epitopes. These epitopes could also be detected in a time and temperature dependent manner. Subsequent studies [114] showed that structural rearrangements seen in the previous study were closely mimicked by three artificial treatments of purified virions. Structural rearrangements of virus exposed to brief incubation at 51°C, treatment with 1–5 mM dithiothreitol, or incubation at pH 5.8 to 6.0 were probed using a panel of MAbs specific for Sindbis virus El and E2 glycoproteins. Infectivity was retained after all three treatments. These observations were interpreted to suggest that Sindbis virions are metastable and can exist in at least two infectious conformations. The authors concluded that these intermediate structures may represent different conformations of a complex pathway that leads to productive infection and was an early indication that infection proceeded through protein structural intermediates induced by virus-cell interactions in the absence of low pH at the cell surface. None of these structural intermediates can be inferred from a single rigid X-ray structure.

A high resolution alphavirus structure was recently reported by Zhang et al. and is shown in Figure 1 [22]. At 4.4 Å resolution, this structure of VEEV was determined by a combination of homology and* de novo* modeling. The final VEEV model was compared to a pseudomodel in which CHIKV E1 and E2 X-ray structures were fit separately into the VEEV model. The results indicate that E2 (residues 1–341) had a higher RMSD value (4.2) than E1 (residues 1–391) with an RMSD of 1.8 Å, representing the difference from identical molecules with an RMSD of 0.0 [115]. The low pH SINV E1-E2 crystal structure was also fit into the VEEV cryo-EM structure (PDB ID: 3MUU, chain A) showing an RMSD of 2.4 for E2 and 2.9 for E1 ectodomain. The transmembrane domain and the E2 endodomain were modeled* de novo*. In addition, the structure of the capsid protein as determined by cryoEM reveals a predicted *α* helix at residues 115–124 that is missing in the capsid structure as revealed by X-ray crystallography. Thus, this cryoEM reconstruction has served to refine the structures of viral components by independently using improved methods to define the protein structures. Advances in single-particle cryo-EM have pushed the limit to near atomic resolution of ~3.3 Å [116–119]. Methods are improving, and soon it should be possible to build a virus model in the absence of structural artifacts without the need to dissociate virions. Subtomogram averaging from* in situ* cryo-EM has the potential of looking at virus proteins without the need for crystallization. Unlike X-ray crystallography, this method ensures that the viral proteins imaged in this manner are in their native conformations. While this method yields lower resolution images than single particle reconstructions, advancements in this technology will undoubtedly improve the resolution.

### 2.4. pH Studies and Viral Penetration

**Figure 10:**
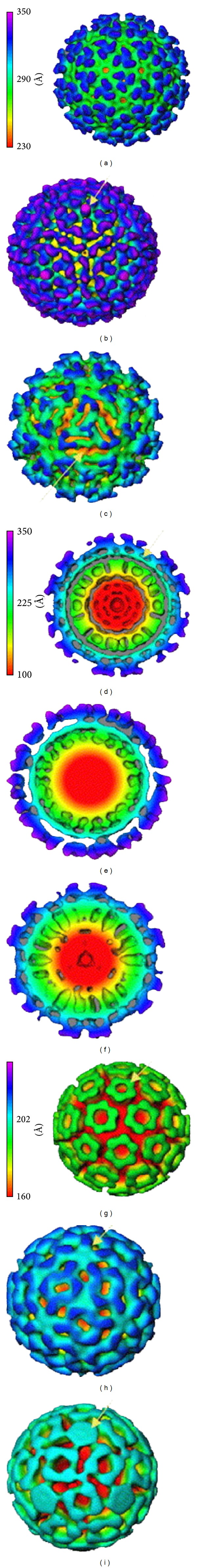
Conformational changes in Sindbis virus after exposure to various pH conditions. The three-dimensional structures of Sindbis virus surface at 28 Å resolution viewed along icosahedral threefold axes ((a)–(c)). Central cross section of Sindbis virus ((d)–(f)). Three-dimensional structure of the Sindbis virus capsid (inside the membrane bilayer) viewed along the icosahedral threefold axes ((g)–(i)). The reconstructions are colored according to a range of radii (key displayed) at different pH conditions ((a), (d), and (g)). Sindbis virus at pH 7.2. ((b), (e), and (h)) Sindbis virus at pH 5.3. ((c), (f), and (i)) Sindbis virus exposed to pH 5.3 (5 min) and returned to pH 7.2. Arrows: (b) protrusion at the fivefold axis; (c) fissure at twofold axis; ((d), (e), and (f)) fivefold axis; (g) region of cross-section occupied by the membrane bilayer. Paredes et al. 2004 [122].

A low pH study of Sindbis virus was undertaken by Paredes et al. in 2004 which hypothesized that low pH triggers the same or similar conformational rearrangements as does contact of the virus with the cell receptor. Sindbis virus treated at low pH was investigated by cryoEM. SVHR is a laboratory strain selected for heat resistance (Sindbis virus heat resistant) [120] which also confers the ability of the virus to produce virus which is ~100% infectious. Infectious, BHK-grown SVHR virus of a particle to pfu ~1 at pH 7.4 was exposed to pH 5.3, returned to neutral pH, and prepared for microscopy. This study revealed that low pH treatment triggered a substantial rearrangement of both E1 and E2 spike proteins and there was a significant formation of nobs of E1 density protruding from each of the 5-fold axes (see arrow in Figure 10(b)). This is the only structure of an alphavirus at a pH which establishes the conditions required for membrane fusion after return to neutral pH. Returning to neutral pH did not restore the native structure and resulted in noninfectious virus. The observation that low pH inactivated alphaviruses had been made early on [121] and can be explained by the inability of the low pH form of the virus to reorganize to the native conformation.

**Figure 11:**
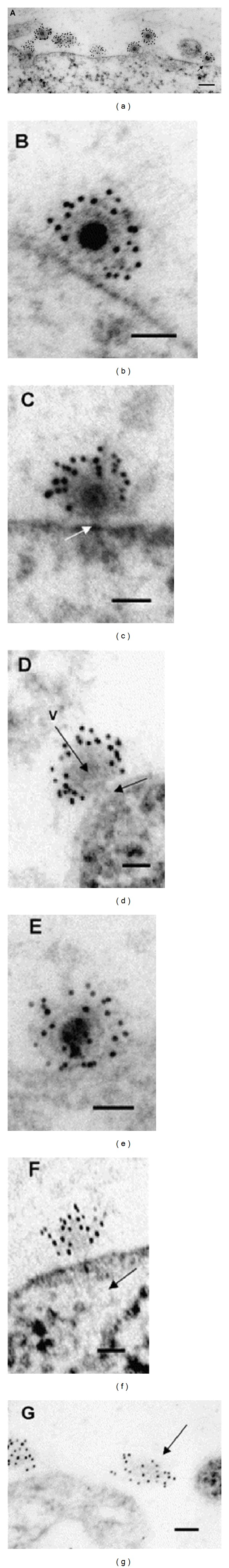
Electron micrographs of thin sections of Sindbis virus-cell complexes at pH 7.2. (a) Low magnification showing “full” and “empty” particles and a particle attached by a pore to the cell surface (arrow). (b) A virion attached to the cell surface before pore formation. (c) A virion with an electron dense core attached to the cell surface by a pore structure (arrow). (d) The pore at the vertex (V) of the protein shell penetrates the cell membrane (arrow). The virion has reduced electron density in the core region. (e) Reorganization of virus RNA into the developing pore. (f) An empty particle with a possible RNA molecule entering the cell (arrow). (g) An empty virion that has lost structure. Magnification scale bar (a) = 1000 Å, all others = 500 Å. Paredes et at. 2004 [122].

That low pH inactivates Sindbis virus in solution is consistent with the observation that treated virus does not return to its infectious conformation [121–124]. For this reorganization of E1 to occur, the virus must be taken to the pH 5.3 threshold since pH changes above this do not affect infectivity of SVHR or establish conditions required for membrane fusion [122, 123]. In another whole virus study using SFV clone pSFV4, low pH structures by Haag et al. only exposed the virus to pH 5.9, not taking the virus through the requisite 5.3 pH for producing the low pH structures and subsequently resulted in very little change in the virus conformation [125]. This is because at the pH required for fusion, concentrated virus samples precipitate. The Paredes et al. study in 2004 proposed a new model for membrane penetration of infectious alphaviruses. Given that the resolution of the low pH structure was 28 Å, it was deemed possible that the knob of density appearing at pH 5.3 may house a proteinaceous pore (Figure 10(b)). This protruding density is ~52 Å in width and ~60 Å in length and is generated from the surrounding “skirt” region at the five-fold axis. This is of sufficient size to house a pore from which RNA could be extruded, roughly 10 Å internal diameter [126]. This hypothesis was substantiated by electron micrographs of infectious Sindbis particles interacting with the host cell surface, creating a pore-like structure through which RNA was seen to extrude (Figure 11). Evidence was also shown that the virion may be interacting via the five-fold axis as suggested by the cryoEM structure. Taken in conjunction with emergence of the new structure of E1 at the five-fold axis and the direct visualization of pore formation at the host cell plasma membrane using EM, it was concluded that virus entry may proceed by an ancient pathway proposed for bacteriophage, direct cell penetration.

A recent study by Cao and Zhang reported an early-stage fusion intermediate of Sindbis virus using cryoEM to reconstruct the low pH intermediate [127]. The strain TE12, which fuses at pH 6.5, was used to conduct this research although this strain has a particle to pfu ratio of ~100. Sindbis virus was then treated to pH 6.4 and the virus was found to retain its *T* = 4 structure. This is surprising since this pH should have induced a global conformational change in both E1 and E2 as was shown previously with SVHR displaying ~100% infectivity [122]. This lack of reorganization of the TE12 strain could be a reflection of a large particle to pfu number or too high a pH to induce the conformational change. Neither virus titer nor particle to pfu ratios were reported for this study. TE12 was then mixed with liposomes at neutral or pH 6.4. At the lower pH, virus was seen to interact with liposomes or vice versa via bridge-like densities spanning the distance between the liposome and the virus. These structures are ~160 Å in length which is a greater span by 10 Å than the soluble low pH structure [127]. When the SVHR strain of Sindbis virus was treated at a pH of 5.2, E1 density at all 12, 5-fold vertices formed knobs of density ~60 Å in length, not the trimeric spikes seen with soluble E1. However, comparisons to the low pH SVHR structure were not made. The authors also report that no specific orientation is required for the virus to bind a target membrane; however, this lack of orientation is determined with liposomes in the absence of the virus receptor and without intact particles. This was not the case with the SVHR study which showed EM evidence of SVHR particles binding to cells at a 5 fold vertex and penetrating the cell at the surface in the absence of low pH [122]. Fusogenic properties of membrane containing virus is often studied using liposomes. Lipid fusion of alpha- and flaviviruses with liposomes is well documented and occurs with very specific admixtures of lipid [124, 128–130]. However, these liposomes are not representative of the composition or structure of the host cell membranes (discussed below). There is direct evidence that Sindbis virus can become noninfectious but retain its fusogenic ability [66, 131], thus separating the E1 fusion function from its infectivity. It is also well documented that alpha and flaviviruses can undergo low pH mediated fusion from within and from without; however, it is possible that the Togaviruses infect cells by a mechanism more similar to that used by polio virus [132] rather than that of influenza [122, 131, 133].

Cell-mediated endosomal uptake of alpha and flaviviruses followed by acidification and membrane fusion with the virus membrane is currently the favored model of alphavirus penetration and entry [134–136]. This mechanism is supported by indirect biochemical evidence and structures of E1 trimers extracted from virus-infected cells [137, 138] or expressed as soluble protein [139–141]. Current evidence of low pH structures for E1 includes studies done using liposomes to extract the proteins from virus during cofloatation [142–144]. While liposomes are useful for studying fusion in other systems in which the virus proteins can be expressed independently such as with flu, these artificial membranes may not be a suitably stringent reagent for the study of virus penetration in the case of alpha- and flaviviruses. This is because (1) no virus receptors are present, (2) high concentrations of cholesterol are required [145–147], and (3) the trimer of E1 has not been seen by any other method other than extraction or expression of E1 followed by liposome interaction. Insects are cholesterol auxotrophs [148] and do not contain the amounts of cholesterol required for liposome fusion [149]. Finally, the fusion of virus with liposomes is a non-leaky process [150] which is not the case with virus infections of host cells [151]. Interestingly, we have also shown that certain clones of mosquito cells derived from Singh's original isolate U4.4 are not susceptible to fusion from without Sindbis virus but are readily infected [123, 152]. Because the details of the fusion model of alpha and flavivirus penetration are predominant in the literature, evidence for direct virus penetration will be further discussed.

### 2.5. Ultrastructural Evidence of Direct Virus Penetration

As early as 1978, Fan and Sefton proposed that virus entry for Sindbis and VSV involved a mechanism which did not require fusion [153]. This evidence gave way to the more popular model of membrane fusion [129, 136]. The* a priori* belief that enveloped virus structures must encode a fusion loop and penetrate cells by fusion, now dominates the field to the exclusion of alternate modes of virus entry. E1 of the Alphaviruses and E of the flaviviruses are referred to as group II fusion proteins [139, 154, 155]. Indirect evidence has been used to develop a model proposing that these types of proteins insert a small fusion loop into the host endosomal membrane after endocytosis and a shift to low pH. The fusion loop is seen in Chikungunya virus [Alphavirus E1 (2ALA)] and flavivirus [Dengue E (1TG8)] crystal structures. Notably, pestiviruses and hepaciviruses, which belong the Family* Flaviviridae*, do not encode a fusion loop [156, 157] and investigators are in search of alternate fusion domains or fusion mechanisms [158]. The Bunyaviridae have been predicted to encode a fusion loop and by homology have been predicted to be class II fusion proteins [159].

A large volume of work has focused on the ability of alpha and flaviviruses to fuse artificial membranes and to elucidate the mechanism of low pH-mediated fusion of virus. The model of infection for these two families posits that membrane-containing viruses infect cells via low pH-mediated fusion within cell endosomes. The membrane-fusion mechanism of virus infection has been studied extensively for the influenza (flu) hemagglutinin and has been shown by direct evidence to form structural intermediates involved in virus penetration [97, 160–162]. Influenza, however, differs significantly from the alpha and flaviviruses in that the structure of the virus is amorphous with the structural proteins associated with large areas of exposed lipid. Additionally, HA and N do not form heterodimeric associations. Unlike influenza, there is no direct biochemical or structural evidence for membrane fusion by arboviruses, and no thermodynamics of the fusion process or the induction of the fusion intermediates.* The ability of alpha and flaviviruses to fuse membranes is not disputed; however, this may not result in virus penetration and infection*.

**Figure 12:**
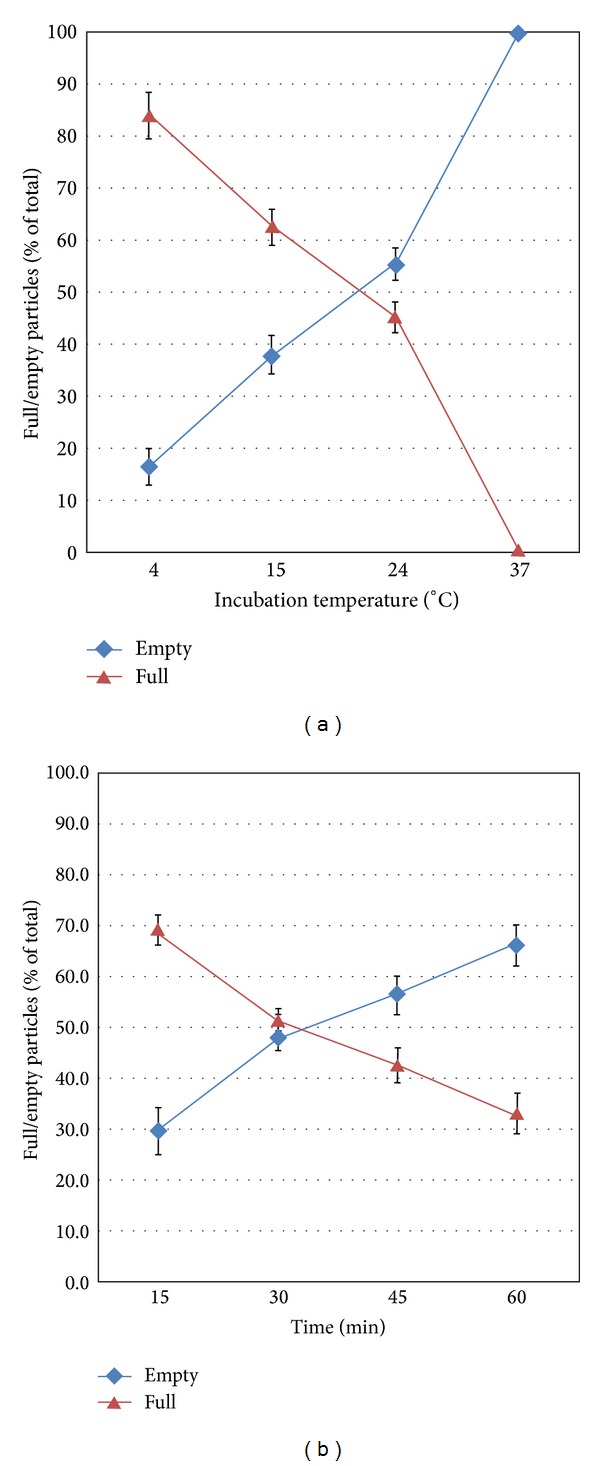
Temperature and time dependence of Sindbis virus genome delivery into BHK cells. (a) Sindbis virus-cell complexes incubated and fixed at 15 min postinfection at several temperatures were analyzed by electron microscopy. The graph shows that, as temperature rises from 4°C to 37°C, there is a progressive increase in the population of empty particles at the cell surface (◆), representing the total population at 37°C, and a decrease in the population of full particles (▲), reaching zero at 37°C. (b) Interaction of Sindbis virus with BHK cells at 4°C for 15 to 60 min. The percentage of empty particles increases from 30% at 15 min to 66.6% at 60 min (◆), and the population of full particles decreases from 70% to 33.4% over the same time period (▲). Data are shown as the means of triplicate samples of two independent experiments. Error bars are standard errors of the means (SEM). From Vancini et al. 2013 [133].

Our work on virus penetration has shown that Sindbis, West Nile virus (WNV), and dengue virus can penetrate cells at temperatures that do not allow membrane fusion [131, 133, 163, 164]. Using direct observation and biochemical methods, we have demonstrated that Sindbis and dengue virus infect cells in a time and temperature dependent manner (Figure 12).

**Figure 13:**
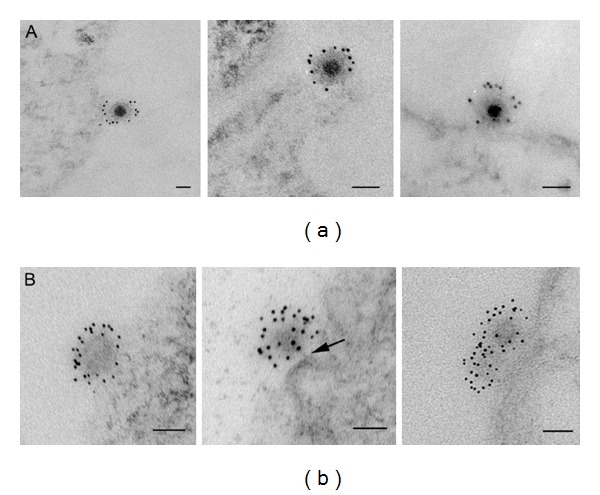
Thin-section electron microscopy of Sindbis virus-cell complexes at pH 7.2. A panel of images obtained by TEM illustrates two representative populations of particles at different incubation temperatures. High-magnification images show examples of electron-dense full particles at the cell surface at 4°C (row (a)) and empty particles with loss of RNA electron density predominantly at 37°C (row (b)). In row (b), middle panel, there is indication of a stalk connecting the virus and the cell (arrow). Bars, 50 nm. From Vancini et al. 2013 [133].

**Figure 14:**
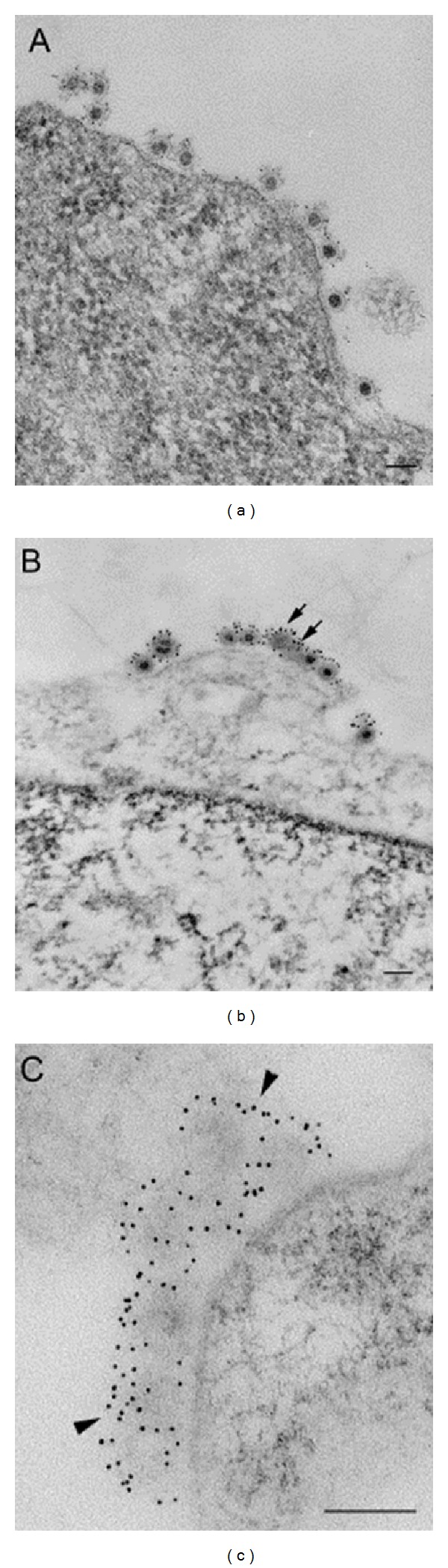
Overview of Sindbis virus-cell complexes. Low magnifications of Sindbis virus-cell complexes at different temperatures. (a) Interaction at 4°C; (b) interaction at 15°C; note the presence of both full and empty (arrows) particles; (c) interaction at 37°C, in which most particles lose their electron density and their well-defined structure (arrowheads). Bars, 100 nm. From Vancini et al. 2013 [133].

Recent data show that Sindbis virus can penetrate mosquito C7-10 cells even more quickly than what has been seen in BHK cells. By 60 min. postinfection at 4°C, 90% of the virus was empty as compared to the 75% seen in BHK cells (see Figure 12(b)). Cultured insect cells contain less cholesterol than mammalian cells and are less viscous at 4°C, which may facilitate the process. The temperature kinetics of this reaction can be fit to Arrhenius plots, suggesting that the process of entry of the RNA into the cell is not force driven and that the energy to form the pore structure likely resides in the virus proteins. The energy of activation is calculated to be 27 kcal/mole. The entry process only requires a membrane potential and is affected by the chemistry of the host cell membrane (Vancini, personal communication). The data show that 70% of Sindbis virions are empty after one hour at 4°C (Figure 13). Sindbis virus carrying a green fluorescent protein will infect BHK cells at 15°C in the absence of fusion, or endocytosis producing fluorescent cells without return through higher temperatures [133]. The obvious implication of these data is that studies in which virus has been allowed to attach on ice for one hour during the infection phase may not have synchronized the infection, as proposed in these studies, but rather allowed infectious particles to be internalized [122, 131, 133, 165], reviewed in [165, 166] (Figure 14). However, the effect of 15°C on formation of the replication complex has not been reported for Sindbis virus but it is possible that synchronization occurs at the level of RNA synthesis.

Strong biochemical support for the model of direct penetration at the cell surface comes from studies showing that cells become permeable to ions and small molecules as they are infected with alphaviruses [126, 151]. Virus infections leave pores on cell membranes [167] that allow the penetration of the cell membrane by small proteins such as the toxin *α*-sarcin (17 kDa) [168]. These results show that pores created in the plasma membrane as entry takes place affect membrane permeability. Fusion, by contrast, is a nonleaky process, does not compromise membranes, and does not leave pores in the membranes [150, 169].

Evidence of direct penetration has been presented for both mammalian and insect cells [165, 166]. This work has led to the alternate model that proposes that alpha and flaviviruses penetrate the host membrane bilayer through host cell triggered rearrangement of E1 or E proteins at a 5 fold axis resulting in the formation of a proteinaceous pore. This pore allows the release of the RNA into the cell cytoplasm, thus initiating the infection process. In the 1994 study by Guinea and Carrasco [170, 171], it was concluded that an ion gradient was required for viral entry of vesicular stomatitis virus, Semliki Forest virus, and influenza virus. This observation has been confirmed for Sindbis virus (Vancini, unpublished). We propose that membrane-containing icosahedral viruses incorporated lipid into their structure as an assembly scaffold allowing the cell exocytic mechanism to process and present maturing structural proteins to the nucleocapsid for envelopment.* This point is crucial for the development of prophylactics for pathogenic arboviruses*.

### 2.6. Native Virus Structure Analyzed Using Small Angle Neutron Scattering

In the paper by He et al. [40], we used small angle neutron scattering to explore the nature of Sindbis virus (alphavirus) particles produced by mammalian and insect cells. This method has the advantage over other methods of observing structure in that lipid and RNA densities are easily detected through a technique called contrast variation [172]. The findings were significantly distinct from what was expected because virus particles from these two hosts have important structural differences. Using virus particles purified in deuterium, a highly concentrated solution of virus suspension was made from virus grown in mosquito C7-10 or mammalian BHK cells. The particles were then analyzed using small angle neutron scattering (SANS), a nondestructive technique [173, 174]. The *R*_*g*_ (radius of gyration) indicated that the BHK-grown virus is less compact than that grown from mosquito cells. The diameter of the BHK grown virus was found to be 689 Å, compared to that of the insect grown virus which was 670 Å. The mass at the center of the BHK particles was less centrally distributed than that seen in the C7-10 virus. It was also determined that while the radial position of the lipid bilayer did not change significantly, the membrane had significantly more cholesterol when the virus was grown in mammalian cells than in insect cells. This property has been shown to affect the virus stability and, with it, infectivity [149]. Distribution of the densities of the particles was modeled using a four shell analysis representing the distribution of the biochemical components of the virus: RNA, capsid protein, lipid with protein, and glycoprotein. Comparing the shell thickness for each virus showed that the outer protein shell was more extended in the mammalian Sindbis virus than that from the insect virus. The SANS data also demonstrated that the RNA and nucleocapsid protein share a closer interaction in the mammalian cell-grown virus than in the virus from the insect host. It is possible that the nucleocapsid structure from mammalian cells is organized more closely to the virus membrane and that the center of the virus is mainly solvent. The biological consequences of the structural differences uncovered by this new technique are not known. It may be that the temperature of assembly may contribute to these differences, given the different biochemical environments. It is of interest to note that Zhang et al. [23] reported a structure of dengue virus at 3.5 Å resolution when grown from C6/36 mosquito cells grown at 33°C (see Figure 3) but did not report a difference in the size of the virus. This may suggest that virus conformation and size may be the result of the host cell biochemistry and not temperature. This will be discussed further.

### 2.7. Temperature Studies and Modeling Crystal Structures into Cryo-EM Reconstructions

**Figure 15:**
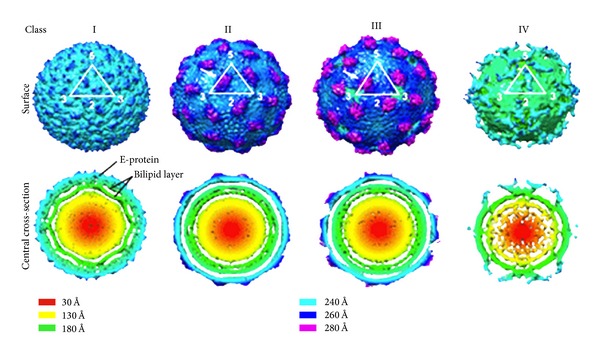
Cryo-EM maps reconstructed from class I to IV particles present in the DENV2 sample incubated at 37°C. The surfaces of the maps (above) and their central cross-sections (below) are colored according to their radii (shown in the lower panel). The white triangle represents an icosahedral asymmetric unit, and the corresponding 2-, 3-, and 5-fold symmetry vertices are indicated. Maps generated from the class I particles are similar to the previous published DENV-2 maps. Class II and III maps showed that the particles in these classes have bigger radii, indicating that the virus had expanded. There are protruding densities on the virus surface between the 5- and 3-fold vertices (white arrows). The class IV map showed very poor density on the E protein layer, indicating that the E protein had lost its icosahedral symmetry. With permission from Fibriansah et al. [175].

Dengue virus particles are structurally classified as (1) mature, (2) partially mature, or (3) immature, depending on the surface morphology seen in cryoEM preparations prior to averaging of the particles [176]. The classification of particles into mature, partially mature, or immature status does not correlate with infectivity; it is estimated that infectious virus contains 30–40% uncleaved preM [84, 177]. Mature dengue has been reported to have a particle diameter of 500 Å [94]. Two recent studies have reported that temperature also affects viral conformation for dengue virus 2 strains 16681 and New Guinea C [175, 178]. In both these DV2 studies, one by Zhang et al. [178] and one by Fibriansah et al. [175], it was determined by cryoEM reconstruction of virus grown in insect C6/36 cells at 28°C then heated to 37°C that virus expanded by 40–50 $\overset{´}{Å}$ compared to the virus remaining at ambient temperature [175]. In each study, both heated and unheated virus was found to have equivalent infectivity, indicating that both virus structures represent infectious intermediates. In the Fibriansah study, several categories of 37°C treated virus particles were seen in the cryoEM images, resulting in sorting of the virus into several classes based on the surface morphology, shown in Figure 15. Class II particles have a smooth surface while Class III display a surface with protrusions. Class IV structures are smaller than the other classes. Class I particles are those grown in mosquito cells at 28°C. In class II viruses, E and M, density was seen to reorganize to produce protruding structures between the 5- and 3-fold vertices. In the class III structures, the protrusions are more extended at the 2-fold axes, and “holes” appear at the strict 3-fold axes. While the structure of the class IV viruses was not further investigated by these authors, particles at 37°C continued to change conformation reorganizing density toward the 5-fold axes until a nonreversible endpoint was achieved. Thus, heating of the class I particles resulted in three different structures. In most icosahedral virus reconstructions from cryoEM, the signal is improved and the noise reduced by imposing icosahedral symmetry on the image data after refinement. For protein density to be visible after imposing this symmetry, the protein structure has to occupy more than 60% of the icosahedrally related positions [179, 180]. Conversely, structures that occupy very few of these positions, such as a portal complex, are averaged out of 3D reconstructions. Thus, while the amount of preM is averaged out during a cryoEM reconstruction, the presence of this protein may still have an important role to play in the structure, infectivity, and immunogenicity of the virus. It was not determined whether this structural difference was a result of a difference in the host environment or a function of the temperature of assembly. Insect host specific biochemistry, which functions optimally at 28°C, may be responsible for assembly of the compact structure which can adopt a less compact form at 37°C. As was discussed above, virus size differences were seen with Sindbis virus from different hosts (mammalian or insect) [40].

The structure by Zhang et al. is similar to the one observed by Fibriansah et al, in which E and M density is seen to move away from the core of the virus after heating insect grown virus to 37°C. The former structure was solved to 35 Å, while the latter had a resolution of 14 Å. In the work by Zhang et al. the same experiment is performed; insect grown virus is heated to 37°C. Both virus structures were found to be equally infectious. These authors reconstructed the heated form to a fit with E protein into trimers, however, unlike Fibriansah who fit E into the traditional herringbone pattern. In the Zhang study, the authors propose that this larger 37°C structure represents the prefusogenic form of the virus that was previously just a predicted structure [94]. In Zhang et al., virus expansion was found to be reversible upon heating to 35°C, and that the end point stable intermediate does not occur until the temperature reaches 37°C, after which the expansion is not reversible. Both studies reported the introduction of holes in the virus surface at the 3′ axes not seen in previous reconstructions.

Both these flavivirus studies speculated that the change in structure may affect the response of the mammalian host by revealing previously unexposed epitopes in virus from insect cells. This is important for mapping neutralizing epitopes* in vitro*, and it was previously not understood how known neutralizing Ab (NAb) anti-DV2 epitopes on the virus bound Ab to sequences that were occluded in the virus structure. This anomaly was explained by proposing that superficially hidden epitopes could be unmasked during thermal flux or “breathing” of the virus [181]. This quandary is now resolved by fitting the proteins into a larger expanded structure formed by heating insect virus to 37°C. However, the E fold in the crystal structure may not be native resulting in difficulty with a fit to the smaller structure. In addition to epitope exposure, what is the biological relevance of these observations? A more compact form of insect virus does not affect an* in vivo* mammalian infection because the more compact 28°C structure will only be exposed to the host for one round of replication, after which the second, 37°C structure will be adopted. Because both compact and expanded forms of the viruses from both hosts are infectious, these virions represent metastable structural intermediates primed for infection, and these two distinct infectious intermediates may represent the optimal form of the structure from its specific host biochemical environment.

One interpretation of these observations is that the mammalian virus is “born” in the prefusogenic-like structure, and the mosquito virus is not. While there is no direct evidence for any function of these predicted intermediate models, mosquito cells were speculated to be infected by virus adopting the fusogenic form via exposure to low pH or contact with the receptor, thus triggering the rearrangement. Again, as in the former study, the Zhang study demonstrated that virus incubated at both temperatures is infectious. This becomes an important point because arboviruses do not always cycle between insect and vertebrate hosts. For mosquito-borne viruses, there exists a “mosquito only” zoonotic cycle in the natural transmission cycle, during which mosquito and virus would only experience ambient temperature [182, 183]. Arboviruses carried by mosquitoes are transmitted from vector to vector horizontally by venereal transmission or vertically as infected eggs. This mosquito cycle is required especially in temperate climates for the virus to over winter. If fusion is required for infectivity, then the “insect only” virus cannot acquire the fusion intermediate in the absence of invoking other mechanisms of structural alteration. Whatever the interpretation, the present data argue that for dengue 2, (1) two infectious dengue virus intermediates are found in nature, one at 28°C and another at 37°C as a result of the temperature of assembly, or (2) virus architecture is determined by host components, which have a temperature component resulting in two structural intermediates, both of which are biologically relevant as was reported in He et al. [40]. Neither of these alternatives need be mutually exclusive. It is of note that the structure reported by Zhang et al. (shown in Figure 3) was made from images of virus grown in insect C6/36 cells at 33°C. At this temperature according to the Zhang et al. study, 50% of the particles should have expanded if temperature were the only contributing factor to particle expansion; however, no increase in particle size was reported [178].

In addition to interpreting the high temperature structures, these papers modeled crystal structures of isolated E into the cryoEM reconstructions. Both studies used E from Tick Borne Encephalitis virus PDB 1SVB [95]. Zhang et al. reconstructed the heated form of the structure to a fit with E protein in trimers of dimers as seen in [94] to 35 Å resolution. Zhang et al. propose that the larger 37°C structure represents the prefusogenic form of the virus that reorganizes the herringbone array into trimers after the virus is exposed to low pH. This structure containing the trimer pattern was previously only a predicted structure [94]. In a separate study, Fibriansah reconstructed the DV2 expanded structure into a 14 Å model by separating the images into three size classes. However, in the Fibriansah study that displayed higher resolution E was modeled into the traditional herringbone pattern in the larger class III structure. The question arises as to which of either of the two models represents a biologically relevant structure. Fab 1A1D-2 [175, 178] and MAb E 111 epitopes are suggested to become more exposed and accessible to add weight to each predicted model. An additional complication to the DV2 expansion story is that Kostyuchenko et al. have reported that dengue 1 and 4 do not undergo heat-related expansion of the virus when insect cell grown virus is exposed to 37°C [184, 185]. If virus expansion is required for infection and the structures are similar in structure and function, it is reasonable to assume that it would happen in all four serotypes. It is suggested, however, that stability of dengue 1 and 4 imparted to these serotypes by surface charges on the virus could explain the lack of conformational changes at higher temperatures. This implies that DV2 is more unstable to heat, but no biochemical evidence was provided. The possibility that these viruses may contain unprocessed preM was not discussed or assessed though preM is known to block infection. It is puzzling that these viruses would not respond to heat but supposedly respond to low pH induced conformations to become infectious.

## 3. Conclusions and Comments

As has been discussed, the structural proteins of the icosahedral arboviruses display a remarkable amount of plasticity. From the same conserved folds, *T* = 3 symmetry is assembled in the flaviviruses, and *T* = 4 for the alphaviruses, representing an approximate 200 Å increase in virion size while the genome lengths are not significantly larger. As discussed for the Sindbis virus C mutant, Y180S/E183G, even larger *T* numbers are possible. Not discussed, but pertinent to this point, is the *T* = 12 structure found in the bunyaviruses that deviate to pleomorphic structures in the other members of the Bunyaviridae. These viruses are also presumed to have arisen from an ancient common fold. The relationship of this adaptation is not understood because the *T* = 12 structure genome size ranges from 10.5 to 22.7 kbp and is composed of a tripartite, single-stranded, negative-sense RNA genome that could theoretically be packaged in a much smaller virion [34]. Plasticity has also been documented in virus assembled from the insect versus the vertebrate host [40]. This was an unexpected outcome but in retrospect not surprising because these host systems are so biochemically and genetically divergent. Alpha- and flaviviruses contain glycoproteins with many cysteines, and more than one disulfide bonded form of these glycoproteins could exist. This is difficult to analyze/detect in the intact virus and, for the most part, has been ignored and the configuration of the crystal structures accepted as native. Additionally, the cryoEM structures of the pH-treated viruses have been interpreted to assume the same structural reorganizations of the soluble E protein in the prefusion conformation which forms a trimer. However, these structures are supportive of indirect evidence for trimerization. Thus, in light of the conflicting evidence for fusion as the mechanism of virus penetration, these data should suggest that the structure of the isolated protein does not always predict the conformation in the intact metastable virus. Even at atomic resolution, reconstruction of an infectious virus cannot lead to definitive evidence of the mechanism of virus penetration without direct biochemical and genetic testing of the model.

It has been a long quest to solve the atomic structure of a membrane-containing virus. As technology improves, atomic details will certainly be resolved. However, there are certain impediments to the process that are not directly related to the technology. First, it has become a canon of some investigators that crystal structures of proteins and their multimeric states represent a native structure. This is a huge assumption for metastable virus proteins because in the absence of control structures or direct functional assays this assumption cannot be tested. Most virus samples also contain a large particle to pfu ratio. The most rigorous experiments would not require the dissolution of the virus particle [186, 187]. This is not to say that structures of proteins are not informative but that the process needs to include a caveat that crystals do not represent the entirety of the many conformations through which macromolecular metastable dynamic viruses proceed to reach the infectious intermediate and then deliver, upon infection, the genomic cargo. This is especially important if the crystal structure is of a single protein of a macromolecular complex that does not naturally exist in solution, such as one that contains membrane proteins. There is a precept that if a protein can be expressed by any expression system, the subsequent folds are representative of the native structure. This assumption may be too simplistic because it is well established biochemically and genetically that assembly of membrane-containing virus requires both host and virus chaperones, including lipids and post translational modifications in a complex and temporal manner to assemble an infectious virus. This exclusive process is not available in any heterologous expression system. An example of this is seen in a study of Sindbis virus E1 in which whole infectious virions were analyzed by mass spectrometry, and it was determined that E1 has cysteines not seen in the alphavirus crystal structure in which all Cys are found as disulfides [66]. The key to these analyses is not the technology employed but rather the starting infectious material. This observation suggests that other problems may exist with the current alphavirus E1 model as was also suggested above for flavivirus E. Very few protein structures are used to build a “fit” into a cryo-EM reconstruction because they are assumed to be interchangeable. If the fit does not work, either the process is abandoned or the protein domains are reoriented. An example of this is found in Fibriansah et al. in which they state that E protein could not be fit into the class II particles due to the lack of structural features required for fitting, although it is possible that the cryo-EM contains data on a structural intermediate; that is, that the structure is expanding in a way that does not fit the current methods, model, or crystal. This implies that there may exist an intermediate structure which adopts an unpredicted structure. These structures are models of a very complicated assembly system. Cryo-EM of heated and low pH-treated viruses is revealing an extraordinary reorganization of the contour of alphaviruses and flaviviruses at ~20 Å resolutions. It seems plausible that, during these conformational reorganizations, structural proteins may proceed through several energetic states until the end point state is achieved. Much more work is required to fully appreciate these intricate structures and their mechanics of assembly and infection.
